# Analyzing the effects of barriers to and facilitators of medication adherence among patients with cardiometabolic diseases: a structural equation modeling approach

**DOI:** 10.1186/s12913-022-07987-3

**Published:** 2022-05-02

**Authors:** Katharina Quaschning, Mirjam Koerner, Markus Antonius Wirtz

**Affiliations:** 1 Institute of Medical Psychology and Medical Sociology, Faculty of Medicine, University of Freiburg, Hebelstraße 29, 79104 Freiburg, Germany; 2 Department of Research Methods in the Health Sciences, Institute of Everyday Culture, Sports and Health, University of Education Freiburg, Kunzenweg 21, 79117 Freiburg, Germany

**Keywords:** Medication adherence, Hypertension, Diabetes mellitus, Questionnaire, Structural equation model

## Abstract

**Background:**

Based on the theoretical model of medication adherence (WHO, 2003), the aims of the study were (1) to develop and test a theory-based multidimensional model for the predictive power of barriers to and facilitators of medication adherence and (2) to identify the mediating effects of barriers to medication adherence on drug-related patient outcomes (barrier “MedAd- “: forget; facilitator “MedAd + ”: regular intake).

**Methods:**

Within a cross-sectional study entitled “Increasing medication adherence to improve patient safety in cardiological rehabilitation (PaSiMed)”, the model was evaluated in structural analytical terms based on data collected online of *N* = 225 patients with cardiometabolic diseases. The revised “Freiburg questionnaire on medication adherence (FF-MedAd-R)" was used to measure the latent constructs (e.g., facilitator: communication; barrier: reservations).”

**Results:**

The structural equation model proved to exhibit an appropriate data fit (RMSEA: .05; CFI: .92). For all first-order facilitators of medication adherence, a high proportion of variance (62–94%) could be explained by the second-order factor “Physician–patient relationship (PPR)”. All paths from “PPR” to the constructs depicting barriers to medication adherence showed significant negative effects. Facilitators (“MedAd + ”) and barriers (“MedAd-”) accounted for 20% and 12% of the variance, respectively, in global items of medication adherence. Whereas “Carelessness” showed a full mediation for “MedAd-”, ‘‘Reservations’’ showed a partial mediation for “MedAd + ”.

**Conclusions:**

“PPR” is an important predictor of patient medication adherence. The results underline the importance of a trustful physician–patient relationship in reducing barriers and enhancing medication adherence.

**Supplementary Information:**

The online version contains supplementary material available at 10.1186/s12913-022-07987-3.

## Introduction

Cardiovascular diseases are the most common cause of death in the industrialized world [1]. Despite the known relationship between cardiometabolic diseases (hypertension [2, 3], diabetes mellitus [4, 5], hyperlipoproteinemia [6, 7]) and elevated cardiovascular morbidity [8, 9] and mortality [10–13], patients often exhibit poor metabolic [5, 13] and blood pressure [14, 15] control. In addition, according to World Health Organization (WHO) estimates, only approximately 50% of all people with chronic diseases take their long-term medications regularly [16]. Increasing medication adherence is thought to reduce cardiovascular morbidity [17] and mortality [10] and to achieve economically significant reductions in health care costs [18].

In contrast to the term “compliance” (representing a more paternalistic view [19]), adherence is defined by the WHO [16] as “the extent to which a person’s behavior-taking medication, following a diet, and/or executing lifestyle changes, corresponds with agreed recommendations from a health care provider” and explicitly incorporates the provider’s responsibility for establishing a good provider-patient relationship as well as for actively involving patients. In general, sufficient adherence is considered to be achieved when at least 80% of medically prescribed medicines are taken regularly [20, 21].

Medication adherence is a complex and dynamic system of different and interrelated influencing factors [16, 22] that can facilitate or hinder the experience and behavior of chronically ill people. As potential causes of poor medication adherence, the WHO lists socioeconomic (e.g. costs), system-related (e.g., insufficient communication), disease-related (e.g., specific problems), therapy-related (e.g., therapy complexity) and patient-related factors (e.g., forget, reservations) [16]. In addition to a good physician–patient relationship (e.g. [22–25]), effective measures to enhance medication adherence include reducing drug complexity [1, 19, 26], promoting self-management (e.g., patient education [27]), and regular contact (e.g., text messages [1, 28]) as part of multidisciplinary care [29].

In addition, it is thought that the mutual exchange of information between physicians and patients (e.g., “Shared decision making (SDM)” [30]) may lead to higher medication adherence [19, 31]. However, existing study results on the effect of “SDM” on the medication adherence of people with cardiometabolic diseases are inconsistent. While in one study a positive effect was observed for a subgroup of patients who had a particularly high need for participation [31], other studies showed no effect [32] or a negative effect [24].

Existing studies have mostly singled out individual aspects of medication adherence and examined them in more detail (e.g., patient-physician communication [22, 23, 33, 34]). Little is known about interrelationships between facilitators of medication adherence (e.g., communication, trust, informedness) and barriers to medication adherence (e.g., reservations, carelessness [24]). To our knowledge, no study was available that examined associations of several facilitating factors of and barriers to medication adherence based on multiple dimensions of the WHO theory model (system, disease, therapy, patient [16]) using a structural equation model.

### Objective of the study

The main objective of this study was to use the WHO theoretical model [16] together with empirical evidence (e.g., [23, 25]) as the basis for developing a theory-based multidimensional model and to test its suitability using empirical data from people with cardiometabolic diseases. The results should contribute to a better understanding of the relationships between different determinants of medication adherence and drug-related patient outcomes (barrier: forget; facilitator: regular intake).

### Study background

The study presented here is part of the project entitled “Increasing medication adherence to improve patient safety in cardiological rehabilitation (PaSiMed)”, which consists of a total of three parts.

In the first part, the “Freiburg questionnaire on medication adherence (FF-MedAd)” for surveying barriers to and facilitators of medication adherence was developed in a theory-based manner on a system-based, disease-based, therapy-based, and patient-based level [16]. The “FF-MedAd” was tested psychometrically in a sample of cardiac rehabilitation patients (*N* = 133) using exploratory factor analysis and reliability analysis [35]. It consists of 2 global items on medication adherence (see Table 1, Rows “MedAd + ”: “I take my medications regularly. “; “MedAd-”: “Sometimes I forget to take my medications.“) and 30 items on 3 facilitators of and 5 barriers to medication adherence (see Table 1, “FF-MedAd”, Column 2). In the second part, interviews on barriers to and facilitators of medication adherence were conducted with cardiac rehabilitation patients (*N* = 22), subjected to content analysis [25], and used to develop the revised “FF-MedAd-R”. One of the key objectives of the revision was complementing the questionnaire by additional items and dimensions that were identified in the interviews as guiding action and being relevant to everyday life (see Table 1, “Interviews”, Column 3). In the third part of the project reported here, “FF-MedAd-R” was used and subjected to confirmatory testing. In its original version, it consists of 86 items, reflecting 14 factors. Of these factors, 10 represent barriers to and 4 represent facilitators of medication adherence (see Table 1, “FF-MedAd-R”, “CFA model” inclusion, Column 4). All items are scored on a 4-point scale ranging from 1 (strongly disagree) to 4 (strongly agree). Higher values correspond to a higher degree of expression of the respective latent constructs or items. A detailed overview of the contents of the items and dimensions can be found in the Additional file 1 (Columns 1–9).

**Table 1:**
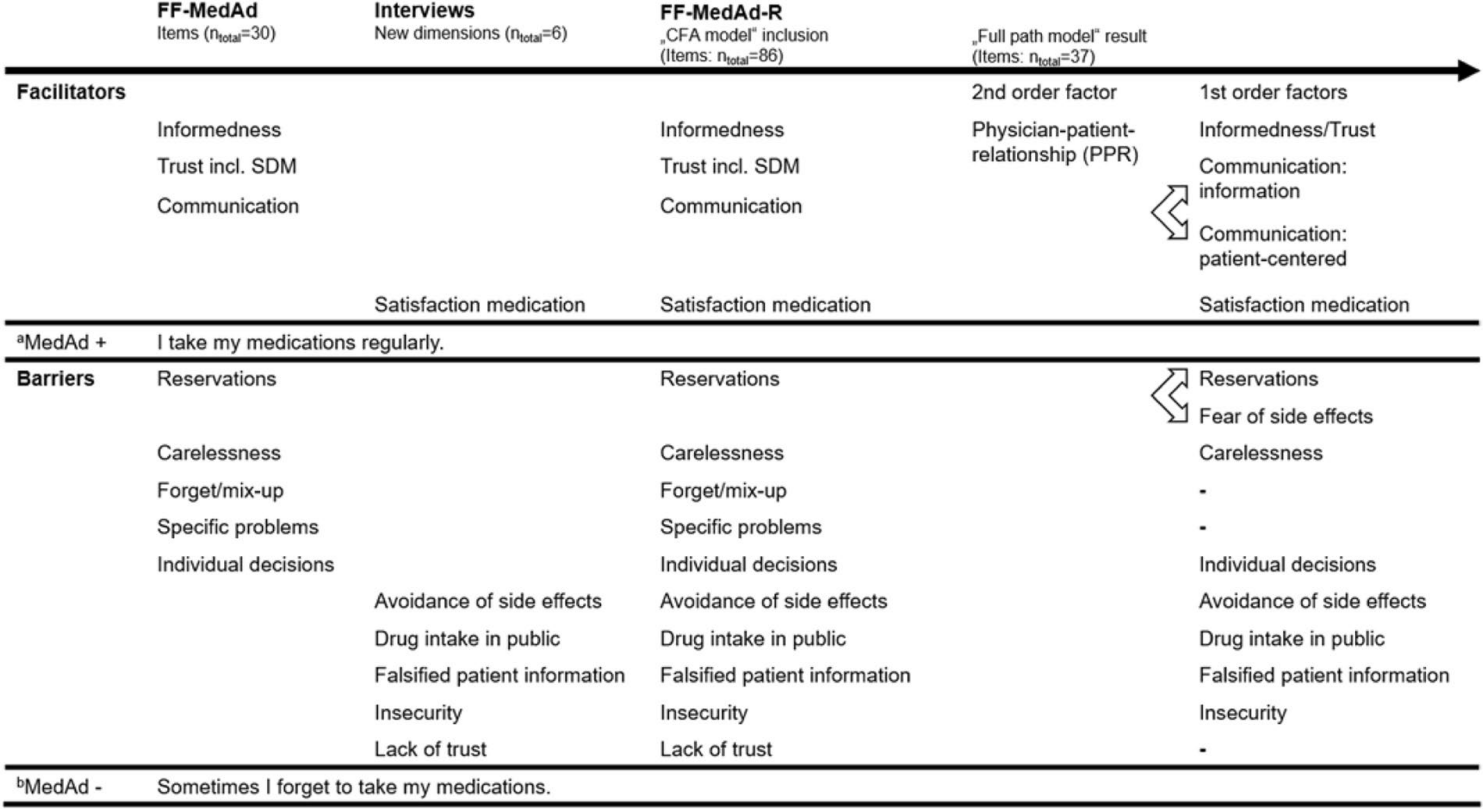
Overview of the questionnaire development process at the dimension level

^a^Global item on facilitators of medication adherence

^b^Global item on barriers to medication adherence

### Research questions and hypotheses

To answer the main research questions, the following hypotheses were formulated regarding the data fit of the complete model and the construct associations:

#### Hypothesis I

The data information of the variables can be adequately modeled by a theory-based structural model.

#### Hypothesis II

The constructs that represent facilitators of medication adherence are independent predictors of “MedAd + ” and “MedAd-”.

#### Hypothesis III

The constructs that are facilitators of medication adherence are predictive of the constructs representing barriers to medication adherence.

#### Hypothesis IV

The effects of the independent variables representing facilitators of medication adherence on the dependent variables “MedAd + ” and “MedAd-” are mediated by the variables representing barriers to medication adherence.

## Methods

### Sample

The data collection took place in a cross-sectional study between September 2020 and February 2021. Participants were patients with cardiometabolic diseases selected via a nationwide self-help organization in Germany [36]. The inclusion criteria for participants were as follows: (1) age ≥ 18 years, (2) long-term medication (> 3 months), (3) hypertension and/or diabetes mellitus and/or hyperlipoproteinemia (self-reported), and (4) confirmation of informed consent. The exclusion criteria for participants with diabetes mellitus were pure insulin therapy because of the need for self-management and self-adjustment as part of the therapy (e.g., intensified insulin therapy). The survey was carried out online using the academic tool “Unipark” [37].

The survey was completed by a total of 234 people. After excluding nine records (*n* = 1: age < 18 years; *n* = 8: pure insulin therapy), the final study sample consisted of 225 patients. A description of the sample can be found in Table 2.

**Table 2:** Characteristics of the sample (*N* = 225)

Age			*M*	*S.D*	*Range*
			62.30	12.5	20–87
Missing			3		
			*Frequencies (n)*		*%*
*Sex*					
Male			130		57.8
Female			93		41.3
Missing			2		0.9
Nationality					
German			220		97.8
Other Nationalities			2		0.9
Missing			3		1.3
Education					
Grammar or high school			113		50.3
Secondary school			64		28.4
Secondary general school			46		20.4
Other			2		0.9
Marital status					
Single			19		8.5
Married/living in partnership			184		81.7
Divorced			8		3.6
Widowed			13		5.8
Missing			1		0.4
Indication^a^					
Hypertension			151		67.1
Diabetes mellitus			168		74.7
Type	1		82		36.4
	2		86		38.3
Treatment regimen					
	Diet/physical exercise		7		3.1
	OAD		21		9.3
	OAD + Insulin		45		20.0
	Insulin		95		42.3
Hyperlipoproteinemia			57		25.3
Type	Hypercholesterolemia		48		21.4
	Hypertriglyceridemia		7		3.1
	I don´t know		1		0.4
	Missing		1		0.4
Coronary heart disease			34		15.1
Heart failure			29		12.9
Heart attack			20		8.9
Stroke			13		5.8
Thyroid diseases			62		27.6
Depression			34		15.1
Other			57		25.3
Participation in patient education^a^					
Yes			154		68.4
Indication		Hypertension	12		5.3
		Diabetes mellitus	141		62.7
		Hyperlipoproteinemia	6		2.7

*M* mean, *S.D.* Standard deviation, OAD Oral antidiabetic drugs

^a^Multiple responses possible

In addition, detailed information on the medications of the sample can be found in Table 3.

**Table 3:** Characteristics on the medications of the sample (*N* = 225)

**Medication (prescribed, daily intake)**	*Frequencies (n)*	*%*
Yes	225	100.0
**Polymedication (prescribed, daily intake)**		
> 5/d [38]	124	55.1
**Medication (prescribed, daily intake)**	*M*	*Range*
Morning	8.9	0–20
Noon	5.1	0–11
Evening	2.3	0–8
At bedtime	1.0	0–8
Total	5.9	1–30
**Medication (prescribed, no daily intake)**		
Yes	47	20.9
No	177	78.7
Missing	1	0.4
**Ad hoc medication (prescribed)**		
Yes	67	29.8
No	156	69.3
Missing	2	0.9
**Self-medication (not prescribed)**		
Yes	100	44.4
No	124	55.2
Missing	1	0.4
If yes^a^:		
Recommended by other people	9	4.0
Homeopathy	17	7.6
Food supplements	36	16.0
Vitamin supplements	53	23.6
Other	36	16.0
**Responsibility for medication**^a^		
Self-medication	213	94.7
Self-medication and family member assisted	12	5.3
Family member assisted	1	0.4
Care services	1	0.4
Other	2	0.9
**Medication plan available (*N* = 225)**		
Yes	101	44.9
No	121	53.8
I´m not sure	3	1.3

*M* Mean

^a^Multiple responses possible

### Data analysis

For the 225 patients included in the analysis, a maximum of 3% missing values in the items of the scales was observed. Prior to the main data analyses, these missing values were imputed by the expectation–maximization algorithm, which estimates missing data using an iterative maximum-likelihood procedure [39–41]. The imputation was performed with the software NORM [42]. For the descriptive statistics of the scales, SPSS 26.0 for Windows software was used [43]. To estimate the multivariate dependencies, structural equation modeling (SEM) was employed [40, 44]. The maximum likelihood estimation procedure implemented in AMOS 26.0 software [45] was used to develop and test all structural models. In the first step [40], a confirmatory factor analysis (CFA) was performed, assuming that all items are distinct indicators of an underlying latent construct, whereby different constructs are allowed to be correlated. The appropriateness of the CFA model was assessed by measures of global and local fit. Measures of global fit indicate whether the empirical associations among the manifest variables are appropriately reproduced by the model [40]. The chi-square provides the strictest form of global model testing [46, 47] because it requires that all the information in the variance–covariance matrix be explained, except for random effects.

Model assessment is usually based on alternative global fit measures. The root mean square error of approximation (RMSEA) indicates the proportion of variance–covariance information not correctly predicted by the model (acceptable model fit: RMSEA ≤ 0.08; good model fit: ≤ 0.05 [40]). In addition, the comparative fit index (CFI) and the Tucker-Lewis index (TLI) were calculated as measures of incremental fit (acceptable model fit: CFI, TLI: ≥ 0.90; good model fit: ≥ 0.95 [40, 48]). Measures of local fit evaluate whether each construct can be reliably estimated from its indicators [47] and whether the constructs within the model are sufficiently distinguishable [47]. To ensure a solid estimation at the construct level, the following indicators of a local fit were applied: the proportion of variance of the indicators predicted by the construct should amount to > 0.40, and the average proportion of variance measured by the construct should be > 0.50 [40, 49]. As criteria for factor reliability, values > 0.60 are accepted as satisfactory [40, 50]. The internal consistency reliability was evaluated using Cronbach's α (adequate: 0.70; good: 0.80; excellent: 0.90 [40]). To check the discriminant validity, the Fornell-Larcker criterion was used, which requires that each construct being more strongly related to its own indicators than to another model construct [49].

In the second step [40], a path model was specified and evaluated using measures of global fit. The significance of the relationships between the exogenous and endogenous latent variables as well as the amount of variance explained in the endogenous variables were examined. To test the mediation hypotheses, the nonparametric BCa bootstrap procedure [51–53] was applied ([95% CI], 1.000 BCa samples).

## Results

### Confirmatory structural modelling (Hypothesis I)

In the first step, a 14-factor measurement model with a total of 86 items (see Additional file 1, Columns 1–3) was specified. The fit measures depicted in Table 4 show that the data were in part insufficiently explained by the model (e.g., TLI; see Table 4, Row “Original CFA model”).

**Table 4:** Measures of global fit for all models estimated

	**χ**^**2**^	**d.f**	**p**	**χ**^**2**^**/d.f**	**TLI**	**CFI**	**RMSEA**
*Thresholds*							
*for acceptable fit*			< *.05*	< *2.5*	> *.90*	> *.90*	< *.08*
*for good fit*				< *2.0*	> *.95*	> *.95*	< *.05*
Original CFA model	6884.45	3524	.000	1.95	.63	.64	.07
Modified CFA model	973.95	589	.000	1.65	.91	.92	.05
Full path model	1108.92	692	.000	1.60	.91	.92	.05

*TLI* Tucker-Lewis index, *CFI* Comparative fit index, *RMSEA* Root mean square error of approximation

For thresholds of acceptable and good fit, see Hair [50] and Kline [40]

A detailed model inspection pointed to three major sources of problems in the model structure: (1) high intercorrelations between the latent variables, which represent facilitators of medication adherence (“Informedness” and “Trust incl. SDM”: *r* = 0.91; “Informedness” and”Communication”: *r* = 0.83; “Informedness” and “Satisfaction medication”: *r* = 0.81; “Trust incl. SDM” and “Communication”: *r* = 0.75; “Trust incl. SDM” and “Satisfaction medication”: *r* = 0.77; “Communication” and “Satisfaction medication”: *r* = 0.57), (2) insufficient item-construct associations (low indicator reliabilities: < 0.40 [40]), and (3) substantial residual correlations between individual items. Thus, the following data and theory-driven modifications were defined. First, the second-order factor “Physician–patient-relationship (PPR)” reflected by the 4 first-order factors dimensions “Informedness”, “Trust incl. SDM”, “Communication” and “Satisfaction medication” was defined. In addition, the constructs “Informedness” and “Trust incl. SDM” were merged into the construct “Informedness/Trust incl. SDM”. Second, modification indices indicated substantial pairwise residual correlations. Furthermore, indicator reliabilities of individual items of the constructs “Reservations” (“res3” [fear of side effects]/ “res8” [fear of interactions]), and “Communication” (“comm4″ [understood and taken seriously]/ “comm5″ [inquiries possible]) proved to be insufficient. Accordingly, the construct “Communication” was divided into the theoretically sound interpretable subconstructs “Communication: information” and “Communication: patient-centered”. Moreover, the items “res3″ and “res8″ were split into the subconstruct “Fear of side effects”. Third, a total of 45 items were sequentially eliminated from different constructs due to double loadings on several factors (*n* = 2) and low indicator reliabilities (*n* = 43; e.g., single item to “SDM”; see Additional file 1, Column “Item Code”, “sdm”). As a result, the scale “Informedness/Trust incl. SDM” was renamed into “Informedness/Trust”. In the fourth step, a total of three dimensions were removed from the model due to insufficient shared item variances: “Lack of trust” (ltrust1–5: indicator reliabilities < 0.40 each), “Specific problems” and “Forget/mix-up” (AVE < 0.50 [40, 49] each). Accordingly, an acceptable to good fit was achieved for all measures (see Table 4, Row “Modified CFA model”). The measures of local fit for the “Modified CFA model” are summarized in Table 5. The threshold for an acceptable fit of indicator reliability was exceeded by 36 of the totals of 37 items, and the *t*-values of all factor loadings were significant. The required threshold values for factor reliability for structural equation models (> 0.60 [40, 50]) were exceeded by all scales. The average variance extracted by each construct from the indicators was 0.54 or higher [40, 49]. In addition, the internal consistency of all scales was adequate to excellent [40].

**Table 5:** Measures of local fit for the “Modified CFA model” (*N* = 225)

			**Item**	**Indicator reliability**	***t*-value of factor loading**	**Cronbachs α**	**Factor reliability**	**AVE**^c^	**Cronbachs α**
***Thresholds for acceptable fit***^b^				> .40	|C.R.|> 2, *p* < .05	> .70	> .60	> .50	> .70
**2**^**nd**^** order factor**	**1**^**st**^** order factors**	**Subfactors**							
Physician–patient relationship	Informedness/Trust		trust1	.78	^_a^	.87	.95	.54	.93
			info4	.54	11.52***				
			info11	.50	11.05***				
			info6	.46	10.46***				
			info9	.43	10.11***				
			info5	.43	10.06***				
			info2	.40	9.74***				
			trust3	.38	9.39***				
	Communication	Communication: information	comm2	.85	^_a^	.90			
			comm3	.76	19.04***				
			comm1	.67	16.80***				
		Communication: patient-centered	comm5	.85	^_a^	.87			
			comm4	.72	15.11***				
	Satisfaction medication		smed8	.61	^_a^	.81			
			smed9	.60	11.45***				
			smed6	.52	10.59***				
			smed4	.41	9.29***				
	Insecurity		ins2	.88	^_a^		.89	.80	.89
			ins3	.74	12.84***				
	Falsified patient information		fals3	.96	^_a^		.94	.80	.94
			fals4	.87	31.56***				
			fals2	.79	25.76***				
			fals1	.57	16.24***				
	Reservations	Reservations	res7	.67	^_a^		.80	.57	.80
			res4	.66	11.96***				
			res9	.40	9.28***				
		Fear of side effects	res3	.71	^_a^		.80	.67	.80
			res8	.64	9.48***				
	Individual decisions		ind4	.52	^_a^		.78	.55	.74
			ind8	.50	8.51***				
			ind1	.48	8.39***				
	Avoidance of drug side effects		ase1	.75	^_a^		.77	.63	.77
			ase2	.55	7.32***				
	Carelessness		carel2	.90	^_a^		.76	.62	.74
			carel1	.52	5.98***				
	Drug intake in public		dip1	.93	^_a^		.78	.65	.77
			dip2	.43	5.10**				

^a^Unstandardized values were set equal to 1 to ensure identifiably

^b^For thresholds of acceptable and good fit, see Hair [50] and Kline[40]

^c^Average variance extracted

^***^p < 0.001

Table 6 (upper off-diagonal values) shows that all latent factors can be sufficiently delimited from one another, as the off-diagonal values (correlations) are always lower than the corresponding line and row values (root AVE) in the diagonal (Fornell-Larcker criterion [49]). No significant correlations were found between the factors (1) “Avoidance of drug side effects” and “Reservations” (*r* = 0.13), “Fear of side effects” (*r* = 0.08), “Insecurity” (*r* = 0.04), “Carelessness” (*r* = 0.12) and “Drug intake in public” (*r* = 0.13), respectively, and (2) “Carelessness” and “Reservations” (*r* = 0.07), “Fear of side effects” (*r* = -0.01), “Insecurity” (*r* = 0.10) and “Drug intake in public” (*r* = 0.07), respectively, and (3) “Falsified patient information” and “Drug intake in public” (*r* = 0.11). All other scales were significantly correlated with each other (see Table 6, lower off-diagonal values).

**Table 6:** Latent construct correlations (upper off-diagonal values), square root of AVE (bold, diagonal) and scale intercorrelations (lower off-diagonal values)

Nr	Factors	1	2	3	4	5	6	7	8	9
1	Physician–patient relationship	**.73**	-.42^e a^	-.29^e^	-.42^e^	-.34^e^	-.39^e^	-.18^c^	-.25^e^	-.24^d^
2	Insecurity	-.36^d b^	**.89**	.37^e^	.50^e^	.35^e^	.44^e^	.13	.13	.19^c^
3	Falsified patient information	-.32^d^	.36^d^	**.89**	.39^e^	.27^e^	.35^e^	.35^e^	.33^e^	.15^c^
4	Reservations	-.37^d^	.44^d^	.31^d^	**.75**	.67^e^	.34^e^	.15	.08	.39^e^
5	Fear of side effects	-.28^d^	.29^d^	.25^d^	.50^d^	**.82**	.27^d^	.10	.02	.22^d^
6	Individual decisions	-.30^d^	.35^d^	.34^d^	.26^d^	.21^c^	**.74**	.51^e^	.29^e^	.29^e^
7	Avoidance of drug side effects	-.14^c^	.04	.35^d^	.13	.08	.36^d^	**.79**	.18^c^	.17^c^
8	Carelessness	-.19^d^	.10	.29^d^	.07	-.01	.20^d^	**.**12	**.79**	.12
9	Drug intake in public	-.22^d^	.16^c^	.11	.32^d^	.15^c^	.18^d^	.13	.07	**.81**

^a^Fornell-Larcker-criterion of discriminant validity: each latent correlation must be lower than both the corresponding row and column value (square root of AVE of each construct)

^b^Interpretation according to product-moment correlation: > .1 weak effect; > .3 moderate effect; > .5 strong effect

^c^Correlations are significant at the level of .05 (2-tailed)

^d^Correlations are significant at the level of .01 (2-tailed)

^e^Correlations are significant at the level of .001 (2-tailed)

**Fig. 1:**
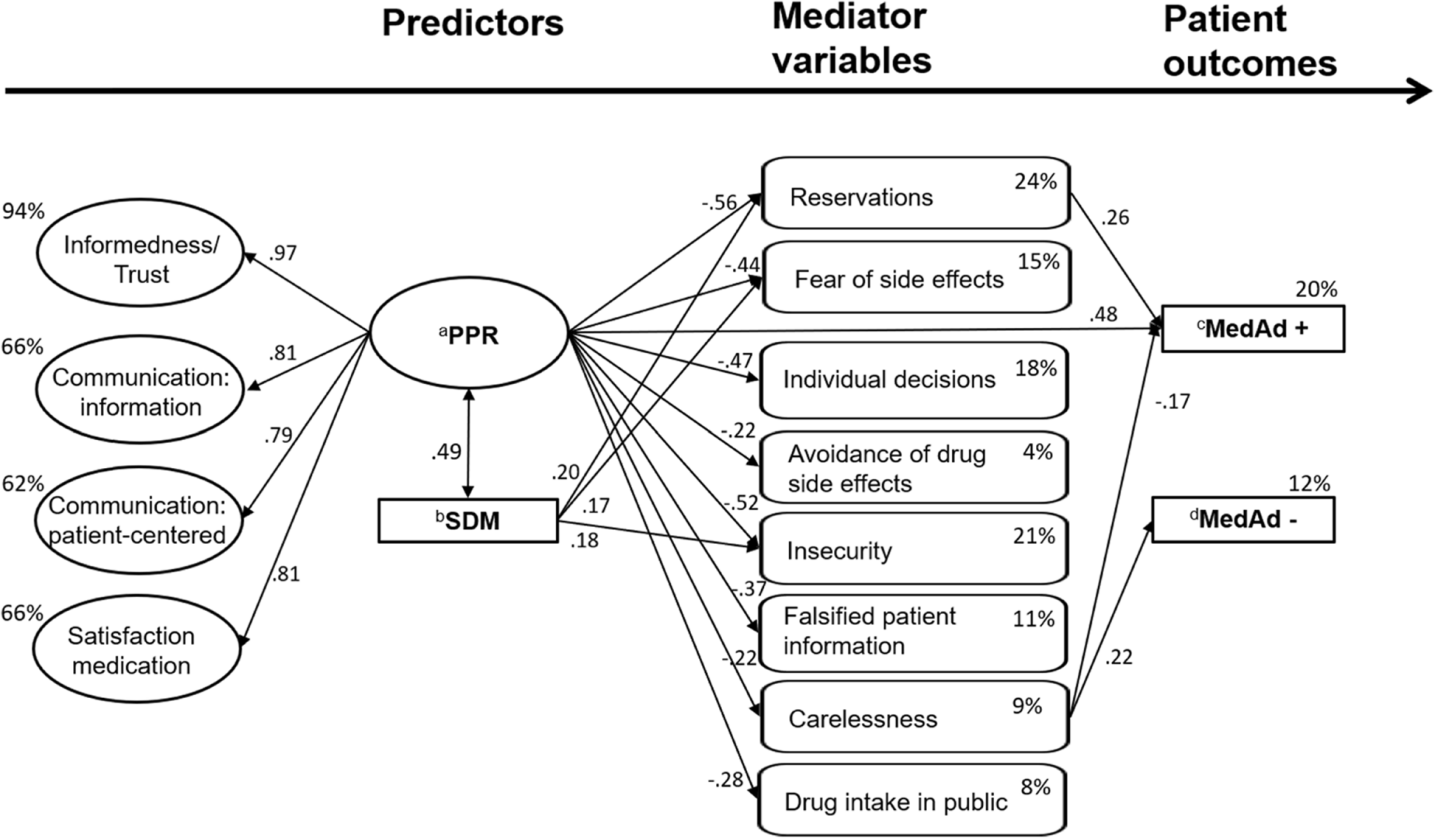
„Full path model “: estimated (only significant) coefficients, mediating effects and percentage of explained variance for the endogenous structural variables. Note: ^a^PPR = Physician patient-relationship; ^b^SDM = Shared decision making; single item: "I have developed my treatment plan together with my physician."; ^c^"MedAd + ": Global item on facilitators of medication adherence: "I take my medications regularly."; ^d^"MedAd-": Global item on barriers to medication adherence: "Sometimes I forget to take my medications". Interpretation according to product–moment correlation (standardized solution): |b|= .1 (weak effect); |b|= .3 (moderate effect); |b|= .5 (strong effect)

As part of the specification of the path model, the second-order factor “PPR” (see Fig. 1, Predictor 1) with its subdimensions “Informedness/Trust”, “Communication: information”, “Communication: patient-centered” and “Satisfaction medication” was interpreted as facilitators of medication adherence, and all other constructs represented barriers to medication adherence (see Fig. 1, Mediator variables). The two global items of medication adherence (see Table 1, Rows “MedAd + ” and “MedAd-”) were added as dependent variables in the path model (see Fig. 1, Patient outcomes). Following the WHO definition of medication adherence [16], the single item of “SDM” was reintegrated in the model (“I have developed my treatment plan together with my physician.”) (see Fig. 1, Predictor 2). An overview of the final dimensions in the “Full path model” can be found in Table 1 (“FF-MedAd-R”, “Full path model” result, Columns 5 and 6). A detailed overview at the item level is shown in the Additional file 1 (Remaining items in the "Full path model", Column 10).

After allowing for the correlation of 8 error terms between factors that represented barriers to medication adherence (e.g., “Reservations”/ “Fear of side effects”; “Individual decisions”/ “Avoidance of drug side effects”) and the correlation of two error terms within the second-order factor “PPR” (“smed8” [medications really help me]/ “Communication: patient-centered”; “info9” [consequences of stopping]/ “Communication: information”), the measures of global model fit indicate a satisfactory to good model fit (see Table 4, Row ‘‘Full path model’’; Hypothesis I).

Figure 1 shows the corresponding model with the resulting parameter estimations of the standardized solution and information on explained variance. Paths were estimated from the predictors to all mediator variables and both outcomes and from all mediator variables to both outcomes. To enable a better overview, only the significant paths are illustrated.

Additionally, the estimates of direct paths are documented in detail in Table 7.

**Table 7:** Direct effects of the “Full path model” (standardized path coefficients of the model)

**Predictors**	**Criteria**	**Hypotheses**^a^	**Beta**	**C.R.**	***P***	**Hypothesis supported?**
H_1_-H_10_: Physician–patient relationship →	MedAd +	+	.48***	4.21	< .001	Yes
	MedAd –	-	-.01	-0.06	.952	No
	Reservations	-	-.56***	-6.03	< .001	Yes
	Fear of side effects	-	-.44***	-4.76	< .001	Yes
	Individual decisions	-	-.47***	-4.82	< .001	Yes
	Avoidance of drug side effects	-	-.22*	-2.47	.013	Yes
	Insecurity	-	-.52***	-6.22	< .001	Yes
	Falsified patient information	-	-.37***	-4.50	< .001	Yes
	Carelessness	-	-.22*	-2.55	.011	Yes
	Drug intake in public	-	-.28**	-3.06	.002	Yes
H_11_-H_20:_ SDM^b^ →	MedAd +	+	-.06	-0.79	.429	No
	MedAd –	-	-.04	-0.54	.591	No
	Reservations	-	.20*	2.55	.011	No
	Fear of side effects	-	.17*	2.02	.043	No
	Individual decisions	-	.15	1.77	.077	No
	Avoidance of drug side effects	-	.04	0.51	.613	No
	Insecurity	-	.18***	2.43	< .001	No
	Falsified patient information	-	.10	1.32	.187	No
	Carelessness	-	-.12	-1.53	.127	No
	Drug intake in public	-	-.01	-0.12	.903	No
H_21_, H_22:_ Reservations →	MedAd +	-	.26*	2.10	.036	No
	MedAd –	+	-.09	-0.72	.473	No
H_23_, H_24:_ Fear of side effects →	MedAd +	-	-.06	-0.56	.577	No
	MedAd –	+	.07	0.60	.551	No
H_25_, H_26_: Individual decisions →	MedAd +	-	-.03	-0.26	.794	No
	MedAd –	+	.16	1.52	.129	No
H_27_, H_28_:Avoidance of drug side effects →	MedAd +	-	-.02	-0.28	.781	No
	MedAd –	+	-.05	-0.55	.579	No
H_29_, H_30_: Insecurity →	MedAd +	-	.15	1.75	.079	No
	MedAd –	+	.02	0.18	.858	No
H_31,_ H_32:_ Falsified patient information →	MedAd +	-	.04	0.57	.570	No
	MedAd –	+	.11	1.40	.161	No
H_33_, H_34:_ Carelessness →	MedAd +	-	-.17*	-2.27	.023	Yes
	MedAd –	+	.22**	2.70	.007	Yes
H_35:_ Drug intake in public →	MedAd +	-	.07	0.86	.392	No
	MedAd –	+	-.02	-0.26	.797	No

*C.R.* Critical ratio

^*^p < 0.05

^**^p < 0.01

^***^p < 0.001

^a^Sign of the assumed relationship

^b^*SDM* Shared decision making; single item: "I have developed my treatment plan together with my physician"

### Prediction of “MedAd + ” and “MedAd- “ by facilitators (Hypothesis II)

For the “Full path model”, it can be summarized that the predictors “PPR” and “SDM” are positively and significantly correlated (*r* = 0.49; C.R. = 5.93; *p* < 0.001). Moreover, “PPR” is an independent predictor of “MedAd + ” (ß = 0.48; C.R. = 4.21; *p* < 0.001) but does not provide any predictive value for “MedAd-” (ß = -0.01; C.R. = -0.06; *p* = 0.952). On the other hand, “SDM” showed no significant relationship with “MedAd + ” (ß = -0.06; C.R. = -0.79; *p* = 0.429) and “MedAd-” (ß = -0.04; C.R. = -0.54; *p* = 0.591) (see Table 7; Hypothesis II).

### Prediction of barriers by facilitators (Hypothesis III)

All direct paths from ‘‘PPR’’ to the constructs representing barriers to medication adherence proved to be significant and pointed in the assumed direction: “Reservations” (ß = -0.56; C.R. = -6.03; *p* < 0.001), “Fear of side effects” (ß = -0.44; C.R. = -4.76; *p* < 0.001), “Individual decisions” (ß = -0.47; C.R. = -4.82; *p* < 0.001), “Avoidance of drug side effects” (ß = -0.22; C.R. = -2.47; *p* = 0.013), “Insecurity” (ß = -0.52; C.R. = -6.22; *p* < 0.001), “Falsified patient information” (ß = -0.37; C.R. = -4.50; *p* < 0.001), “Carelessness” (ß = -0.22; C.R. = -2.55; *p* = 0.011) and “Drug intake in public” (ß = -0.28; C.R. = -3.06; *p* = 0.002). In addition, “SDM” only influenced “Reservations” (ß = 0.20; C.R. = 2.55; *p* = 0.011), “Fear of side effects” (ß = 0.17; C.R. = 2.02; *p* = 0.043) and “Insecurity” (ß = 0.18; C.R. = 2.43; *p* =  < 0.001) to a small degree (see Table 7). The R^2^ value [40] of the constructs representing barriers to medication adherence ranged from 0.04 (“Avoidance of drug side effects”) to 0.24 (“Reservations”) (see Fig. 1).

The construct “Carelessness” significantly predicts “MedAd-” (ß = 0.22; C.R. = 2.70; *p* = 0.007) and “MedAd + ” (ß = -0.17; C.R. = -2.27; *p* = 0.023). Moreover, “Reservations” significantly predicts “MedAd + ” (ß = 0.26; C.R. = 2.10; *p* = 0.036) but does not provide any predictive value for “MedAd-” (ß = -0.09; C.R. = -0.72; *p* = 0.473).

Furthermore, “PPR” significantly predicts (each p < 0.001) the following first-order dimensions strongly: ß_PPR → Communication: information_ = 0.81; C.R. = 11.55; ß_PPR → Communication: patient-centered_ = 0.79; C.R. = 10.76; ß_PPR → Satisfaction medication_ = 0.81; C.R. = 9.65. As a supplement, “Informedness/Trust” could not be tested for significance (reference variable, set equal to 1). In total, 94% of the variance of “Informedness/Trust”, 62% of the variance of “Communication: patient-centered” and 66% of the variance in “Communication: information” and “Satisfaction medication” was explained by “PPR”. In total, the final model accounted for 20% of the variance in “MedAd + ” (R^2^ = 0.20) and 12% of the variance in “MedAd-” (R^2^ = 0.12) (see Fig. 1).

### Mediating effects of barriers on “MedAd + ” and “MedAd-” (Hypothesis IV)

Regarding the indirect paths, for the construct “Carelessness”, complete mediation was demonstrated for the prediction of the variable “MedAd-” (beta = -0.05 [-0.230; -0.013]) and partial mediation was demonstrated for the construct “Reservations” for the prediction of the variable “MedAd + ” (beta = -0.15 [-0.465; -0.023]) by “PPR”. For “SDM”, however, no mediating effects were found.

### Descriptive statistics

Descriptive statistics for all scales of the “Full path model” are shown in Table 8. “Physician–patient-relationship” (*M* = 3.47) proved to be “high” from the patient perspective on average. In contrast, “Carelessness” (*M* = 1.10), “Falsified patient information” (*M* = 1.21), “Individual decisions” (*M* = 1.38), “Avoidance of drug side effects” (*M* = 1.45) and “Drug intake in public” (*M* = 1.67) were evaluated from their perspective as “low”, and “Insecurity” (*M* = 2.04), “Reservations” (*M* = 2.15) and “Fear of side effects” (*M* = 2.23) were evaluated as “moderate”. The appropriateness of the assumption of a multivariate normal distribution (skewness < 3 [40]) was shown for 8 of the 9 model variables.

**Table 8:** Descriptive statistics for all scales (*N* = 225) of the “Full path model”

Factor	Theoretical range	*M*	*S.D*	*Skewness*^c^
Physician–patient relationship^a^	1–4	3.47	.46	-1.28***
Reservations^b^	1–4	2.15	.77	.39*
Fear of side effects^b^	1–4	2.23	.82	.25
Individual decisions^b^	1–4	1.38	.56	1.45***
Avoidance of drug side effects^b^	1–4	1.45	.73	1.47***
Insecurity^b^	1–4	2.04	.81	.45**
Falsified patient information^b^	1–4	1.21	.47	2.52***
Carelessness^b^	1–4	1.10	.31	3.63***
Drug intake in public^b^	1–4	1.67	.81	1.32***

*M* Mean, *S.D.* Standard deviation

^a^High value correspond to a ‘‘good’’ Physician–patient relationship

^b^High values correspond to a high degree of barriers to medication adherence

^c^*p < 0.05, **p < 0.01,***p < 0 .001

## Discussion and conclusion

### Discussion

Based on the theoretical model of medication adherence [16] and empirical findings (e.g., [23–25, 27, 35]), a structural equation model was developed to examine the associations of several barriers to and facilitators of medication adherence in a sample of people with a high risk of cardiovascular disease.

The latent constructs operationalized in “FF-MedAd-R” questionnaire formed the basis for the specification of the model. After excluding the latent constructs “Forget/mix-up”, “Specific problems” and “Lack of Trust”, all other dimensions in the SEM could be adequately modeled by the empirical data (see Hypothesis I).

In accordance with other empirical findings, patient-centered communication by the physician, sufficient information and trust corresponded to an increase in medication adherence (e.g., [22–25]). To the best of our knowledge, there have been no empirical findings on patient satisfaction with medication as part of a higher-level construct of PPR. Future research should examine the stability of this finding and the dependence on patient characteristics in more detail.

In contrast, no direct association was shown for forgetting to take medications (“MedAd-”) as a central barrier to medication adherence [25] by “PPR”. In agreement with other study results, forgetting to take medications tends to be unintentional (e.g., [16]) and may be more effectively reduced by other interventions (e.g., establishment of routines, targeted reminder strategies, medication plan [35]). In addition, there was no direct effect of “SDM” to either global item of medication adherence (see Hypothesis II).

Furthermore, it could be shown that a good physician–patient relationship is substantially associated with lower patient-related barriers to medication adherence. Conceivably, a sufficient degree of informedness might reduce reservations, fear of side effects, or insecurities based on contradictory information and might lead to a reduction in independent dose adjustments. Patients who trust their physicians might be less likely to conceal treatment-relevant information from them. In accordance with other study findings [31, 32], we found little evidence of an association between “SDM” and medication adherence. In addition, a positive effect of “SDM” was found only for the dimensions “Reservations”, “Fear of side effects” and “Insecurity”. In accordance with existing study results [31] it is conceivable that people who are confused, such as by inconsistent information, are more likely to participate in the treatment process (seeHypothesis III).

The effect of “PPR” on “MedAd-” proved to be completely mediated by “Carelessness”. This means that a good physician–patient relationship can contribute to reducing careless behavior. In turn, a low level of careless behavior contributes to an increase in regular medication intake.

Furthermore, the effect of “PPR” on “MedAd + ” proved to be partially mediated by “Reservations”. This means that a good physician–patient relationship may not only have a direct positive effect on regular medication intake but also could directly contribute to reducing reservations. Fewer reservations, in turn, may promote the regularity of medication intake. All other mediation hypotheses had to be rejected (see Hypothesis IV).

As a supplementary result, it was noticed that only 6 people with hyperlipoproteinemia and 12 people with hypertension in the study sample stated that they had participated in structured patient education programs in the past (see Table 2), and only approximately 45% of the respondents reported having a written medication plan (see Table 3). This indicates that the prevention potential (e.g., of patient education [25, 27]), especially for people with hypertension and HLP, is not yet sufficiently exploited in the German health care system.

### Limitation of this study

The “FF-MedAd-R” questionnaire was used for the first time. Online testing ensured standardized completion conditions. Although we did check the quality criteria, a comprehensive psychometric validation has yet to be conducted (e.g., criterion validity).

All data were self-reported by the respondents. There was no review of the information, e.g., about electronic medical records. In addition, further biases cannot be ruled out (e.g., selection bias, recall bias, information bias, social desirability).

The data stem from a cross-sectional sample. Since Intervention studies can only provide information on causal effects, no causal interpretation of the relationships found in the SEM prediction model is allowed. Although this study examined the associations between multiple barriers and facilitators in predicting medication adherence, causal conclusions should be considered against the background of the theoretical model. There are a number of other influencing factors [31] that could not be examined in this study.

In summary, many items and three dimensions had to be eliminated from the models to reach an acceptable global data fit. Hence, the model definition was to a considerable extent data-driven. Although this modification did not lead to chances in the general definition of the corresponding constructs, it was partly exploratory in nature and requires cross-validation.

In addition, many hypotheses were tested in one data set. This may have led to the problem of alpha error inflation.

The SEM approach applied here did not control for moderator variables and potential sociodemographic or indication-related confounders. Such influences need to be examined in further multigroup analyses of the structural model in larger studies.

The inclusion of additional objective measures would have been useful, especially for the dependent variables "MedAd + " and "MedAd- “.

Using a nationwide self-help organization for data acquisition might have led to limitations in both the representativeness of the sample and the generalizability of the results. Persons unfamiliar with the use of digital media (e.g., elderly people) are likely underrepresented. In contrast, persons with a high education level and those with German citizenship are overrepresented in this sample. It is conceivable that the members of self-help groups are particularly motivated people who may be better informed about a more intensive confrontation with their disease and, therefore, tend to exhibit more medication-adherent behavior.

## Conclusion

In summary, the results of this study make an important contribution to theory development and can be used as the basis for developing future interventions in the field of medication adherence in patients experiencing from cardiometabolic diseases. The questionnaire “FF-MedAd-R” has satisfactory reliability and validity (content and construct validity). It is a useful instrument that can be used in everyday clinical practice to measure self-reported medication adherence at multiple levels. The physician–patient relationship was found to be the strongest predictor. The presented findings of construct association should be analyzed in a more differentiated manner. Furthermore, intervention studies should be conducted to critically examine causal inferences and to utilize the findings to improve medication adherence in the clinical practice of patient care.

### Practice implications

The questionnaire “FF-MedAd-R” is a multifaceted, time-efficient tool for medical practice that can be used to identify problems in medication adherence in patients experiencing from cardiometabolic diseases on multiple levels and to derive targeted measures. For example, the questionnaire can be used as a template for asking about problems (e.g., fear of side effects, forget) or patient needs (e.g., wish for information) before or during a physician–patient conversation. Problems with the medication could be recorded (e.g., with the aid of a supplementary checklist) and considered for future prescriptions in order to ensure the effectiveness of prescribed medication and increase patient satisfaction with medication. Communication training for physicians can help them learn suitable communication strategies and make better use of the prevention potential of more deliberate relationship management. Patients should be made aware of structured education programs as an option to increase their knowledge and empowerment skills.

When designing future interventions, the concerns and perspectives of patients, physicians, and all other members of the interprofessional teams should be considered in terms of needs, potential for improvement, feasibility, acceptance, and relevance to everyday life.

## Supplementary Information

**Additional file 1.**

## Data Availability

All data generated or analyzed during this study are included in this published article and its supplementary information files.
