# Supplementary material for: Analyzing the effects of barriers to and facilitators of medication adherence among patients with cardiometabolic diseases: a structural equation modeling approach
Source: BMC Health Serv Res. 2022 May 2;22:588. doi: 10.1186/s12913-022-07987-3 (PMC9063142; doi:10.1186/s12913-022-07987-3)
Supplement: Supplementary file 1 — Additional file 1. [file 12913_2022_7987_MOESM1_ESM.docx]

**Additional file 1**

Summary of constructs and items of the "FF-MedAd-R" questionnaire of medication adherence (barriers/ faciliators)

|  |  | **Items included in the "CFA-model" (n_total_=86)** | **Background of the item development** | | | | | | **Remaining items in the "Full-path-model" (n_total_=37)** |
| --- | --- | --- | --- | --- | --- | --- | --- | --- | --- |
| **Construct** | **Item Code** | **Item**^h^ | **O**^a^ | **OM**^b^ | **OMW**^c^ | **OW**^d^ | **I**^e^ | **A**^f^ | **Final**^g^ |
| **Faciliators of medication adherence** | | | | | | | | | |
| **Informedness** | **Sufficient informedness as a key foundation for successful disease and/or medication management** | | | | | | | | |
|  | info1 | I know why I take each of my medications. | x |  |  |  |  |  |  |
|  | info2 | I am familiar with the dosing of my medications. | x |  |  |  |  |  | x |
|  | info3 | I am familiar with when to take my medications. | x |  |  |  |  |  |  |
|  | info4 | I feel sufficiently informed about my conditions. |  |  | x |  |  |  | x |
|  | info5 | I feel sufficiently informed about the effects of my medications. |  |  |  |  | x |  | x |
|  | info6 | I feel sufficiently informed about possible side effects of my medications. |  |  |  |  | x |  | x |
|  | info7 | I know the names of my medications. |  |  |  |  | x |  |  |
|  | info8 | I have been sufficiently informed about how long I need to take the medications. |  |  |  |  | x |  |  |
|  | info9 | I have been sufficiently informed about the possible consequences of stopping my medications. |  |  |  |  | x |  | x |
|  | info10 | I have been sufficiently informed about whether there are any alternatives to my medications. |  |  |  |  | x |  |  |
|  | info11 | I understand all the information on the medications I have been prescribed by my physician. |  |  |  |  | x |  | x |
| **Trust incl. SDM** | **Patient trust (incl. in the provider, medications) and patient involvement in the treatment processes** | | | | | | | | |
|  | trust1 | I believe that all medications my physician prescribes me actually do help me. | x |  |  |  |  |  | x |
|  | trust2 | I generally trust medications. | x |  |  |  |  |  |  |
|  | trust3 | I trust my physician’s treatment. | x |  |  |  |  |  | x |
|  | trust4 | I have always felt physicians to be very helpful. |  |  |  |  | x |  |  |
|  | sdm | I have developed my treatment plan together with my physician. | x |  |  |  |  |  |  |
| **Communication** | **Physician requirements for ensuring successful communication about medications (incl. patient information, patient-centered attitude)** | | | | | | | | |
|  |  | When my physician talks with me about my medications... | | | | | | | |
|  | comm1 | ...he or she communicates that it is important for me to take my medications regularly. | x |  |  |  |  |  | x |
|  | comm2 | ...I feel informed about how to take the medications properly. | x |  |  |  |  |  | x |
|  | comm3 | ...I feel informed about the effect of the medications. | x |  |  |  |  |  | x |
|  | comm4 | ...I feel understood and taken seriously by my physician. | x |  |  |  |  |  | x |
|  | comm5 | ...I can ask questions about my medications. | x |  |  |  |  |  | x |
| **Satisfaction**  **Medication** | **Medication-related patient satisfaction (incl. positive attitudes, experiences)** | | | | | | | | |
|  | smed1 | I am satisfied with the medications I have to take at the moment. |  |  |  |  | x |  |  |
|  | smed2 | I have always felt that medications were very helpful. |  |  |  |  | x |  |  |
|  | smed3 | I have had many positive experiences with medications. |  |  |  |  | x |  |  |
|  | smed4 | My medications help me improve my parameters. |  |  |  |  | x |  | x |
|  | smed5 | My medications help me feel healthier. |  |  |  |  |  | x |  |
|  | smed6 | My medications help me easily manage my everyday life. |  |  |  |  |  | x | x |
|  | smed7 | I can rely on my medications. |  |  |  |  |  | x |  |
|  | smed8 | I am very happy that there are medications that really help me. |  |  |  |  |  | x | x |
|  | smed9 | My medications help me stay healthy and feel good. |  |  |  |  |  | x | x |
|  | smed10 | Without my medications, I would feel much worse. |  |  |  |  |  | x |  |
| **Barriers to medication adherence** | | | | | | | | | |
| **Reservations** | **Patient reservations regarding the medication to be taken (incl. preference for homeopathy, fear of possible consequences)** | | | | | | | | |
|  | res1 | I would prefer an alternative medicine treatment (e.g., homeopathy) over treatment with medications. | x |  |  |  |  |  |  |
|  | res2 | I generally want to take as few chemical products as possible, e.g., in the form of medications. |  | x |  |  |  |  |  |
|  | res3 | I am worried about possible side effects of medications. | x |  |  |  |  |  | x |
|  | res4 | I am worried about medications harming me in the long term. | x |  |  |  |  |  | x |
|  | res5 | I believe that I have to take too many medications. | x |  |  |  |  |  |  |
|  | res6 | It is very important to me to take few medications in the long term. |  |  | x |  |  |  |  |
|  | res7 | I am afraid that medications could damage my organs (e.g., my kidneys). |  |  |  |  | x |  | x |
|  | res8 | I am worried about possible interactions of my medications. |  |  |  |  | x |  | x |
|  | res9 | I have taken my medications for far too long already. |  |  |  |  | x |  | x |
|  | res10 | I believe that the dose of my medications is sometimes too high. |  |  |  |  | x |  |  |
| **Carelessness** | **Careless handling of medically prescribed medications by patients (incl. in case of deviations from the routine or freedom from symptoms)** | | | | | | | | |
|  | carel1 | Sometimes I cannot find my prescriptions. |  | x |  |  |  |  | x |
|  | carel2 | Sometimes I cannot find my medications. |  | x |  |  |  |  | x |
|  | carel3 | On vacation, I have problems with taking medications more commonly than at other times. |  | x |  |  |  |  |  |
|  | carel4 | On the weekends, I have problems with taking medications more commonly than at other times. |  |  |  |  | x |  |  |
|  | carel5 | Sometimes, I take medications for conditions with lower readings less seriously than those for other conditions. |  |  |  |  | x |  |  |
|  | carel6 | Sometimes, I tend to skip medications for conditions I do not notice, but not medications for other conditions. |  |  |  |  | x |  |  |
| **Forget/ mix-up** | **Forgetting, confusing, or mixing up medications (incl. times of administration, individual strategies)** | | | | | | | | |
|  | fomi1 | Sometimes I mix up my medications. | x |  |  |  |  |  |  |
|  | fomi2 | Sometimes I mix up the times to take different medications. |  | x |  |  |  |  |  |
|  | fomi3 | I have to concentrate very hard to keep from confusing my medications. |  |  |  |  | x |  |  |
|  | fomi4 | Sometimes I forget to get new medications on time. |  |  |  |  | x |  |  |
|  | fomi5 | Sometimes I increase the dose of my medications after I forgot to take them. |  | x |  |  |  |  |  |
|  | fomi6 | A regular daily routine helps me remember to take my medications. |  |  |  |  | x |  |  |
| **Specific**  **problems** | **Medication-related and therapy-related problems (incl. texture, handling, complexity of treatment)** | | | | | | | | |
|  | sp1 | Sometimes I have a hard time splitting my tablets. |  | x |  |  |  |  |  |
|  | sp2 | Sometimes I have a hard time getting new medications on time. |  | x |  |  |  |  |  |
|  | sp3 | Sometimes I have a hard time taking medications out of the packaging. |  | x |  |  |  |  |  |
|  | sp4 | Sometimes I do not take medications because they taste bad (e.g., bitter). |  |  | x |  |  |  |  |
|  | sp5 | Sometimes I do not take medications because I have a hard time swallowing them (e.g., due to their size, no water available). |  |  | x |  |  |  |  |
|  | sp6 | I think my therapy requires a lot of effort. |  |  | x |  |  |  |  |
|  | sp7 | I have a hard time telling my medications apart (e.g., similar appearance/name). |  |  |  |  | x |  |  |
|  | sp8 | When the names of my standard medicines change, I get confused. |  |  |  |  | x |  |  |
| **Individual**  **decisions** | **Medication-related decisions (e.g., dose reduction, withdrawal trial) made by patients independently without consulting the treating physician** | | | | | | | | |
|  | ind1 | If I believe that I have side effects from a medication, I sometimes stop taking the medication. | x |  |  |  |  |  | x |
|  | ind2 | I take my medication only if I believe it to be necessary. | x |  |  |  |  |  |  |
|  | ind3 | I have difficulty organizing the taking of medications in my everyday life. | x |  |  |  |  |  |  |
|  | ind4 | If I believe that I have side effects from my medications (e.g., nausea), I reduce the dose without consulting my physician. |  | x |  |  |  |  | x |
|  | ind5 | I have developed a sense for when I need a certain medication and when I do not. |  |  |  |  | x |  |  |
|  | ind6 | I take my medications at the times that work well in my daily routine. |  |  |  |  | x |  |  |
|  | ind7 | Sometimes I stop taking medications when I feel better. |  |  |  |  | x |  |  |
|  | ind8 | Sometimes I skip medications on certain days when I do not feel good. |  |  |  |  | x |  | x |
|  | ind9 | Sometimes I take medications later if I forgot them. |  |  | x |  |  |  |  |
| **Avoidance of side effects** | **Independent dose adjustments by patients due to feared restrictions in everyday life (e.g., from diuretics)** | | | | | | | | |
|  | ase1 | Sometimes I skip certain medications (e.g., diuretics) on certain days because they would make it difficult to engage in certain everyday activities (e.g., shopping, leisure activities). |  |  |  |  | x |  | x |
|  | ase2 | Sometimes I reduce the dose of certain medications (e.g., diuretics) on certain days because they would make it difficult to engage in certain everyday activities (e.g., shopping, leisure activities). |  |  |  |  | x |  | x |
| **Drug intake**  **in public** | **Discomfort with taking medications or performing therapy-related measures in public** | | | | | | | | |
|  | dip1 | I find it uncomfortable to take my medications in the presence of other people. |  |  |  | x |  |  | x |
|  | dip2 | I find it uncomfortable to perform necessary treatment-related measures in the presence of other people (e.g., checking my blood sugar). |  |  |  |  | x |  | x |
| **Falsified**  **patient**  **information** | **Patient concealment of treatment-relevant information from the treating physician** | | | | | | | | |
|  |  | I sometimes conceal information from my physician (e.g., side effects, skipping of medications) because I worry about... | | | | | | | |
|  | fals1 | ... being prescribed even more medications. |  |  |  |  | x |  | x |
|  | fals2 | ... having to stay in the hospital for an even longer time. |  |  |  |  | x |  | x |
|  | fals3 | ... having to spend even more effort taking medications. |  |  |  |  | x |  | x |
|  | fals4 | ... the medication regimen becoming even more complicated (e.g., taking medications more times per day). |  |  |  |  | x |  | x |
| **Insecurity** | **Insecurity of patients caused by inconsistent information** | | | | | | | | |
|  | ins1 | Sometimes I am insecure due to contradictory information on medications, e.g., from magazines or the Internet. |  |  |  |  | x |  |  |
|  | ins2 | Sometimes I am insecure due to contradictory information from different physicians regarding medications. |  |  |  |  | x |  | x |
|  | ins3 | Sometimes I am insecure due to contradictory information from different physicians regarding treatment options. |  |  |  |  | x |  | x |
| **Lack of trust** | **Lack of patient trust in physicians, medications (e.g., placebos), or the pharmaceutical industry** | | | | | | | | |
|  | ltrust1 | I view physicians very critically. |  |  |  |  | x |  |  |
|  | ltrust2 | I view the pharmaceutical industry very critically. |  |  |  |  | x |  |  |
|  | ltrust3 | I have had bad experiences with physicians. |  |  |  |  | x |  |  |
|  | ltrust4 | I think my physician is influenced by the pharmaceutical industry. |  |  |  |  | x |  |  |
|  | ltrust5 | I believe that my physician has prescribed me medications despite knowing that they are ineffective. |  |  |  |  | x |  |  |

^a^O: Original items of "FF-MedAd" questionnaire, used unmodified in "FF-MedAd-R" questionnaire (n_total_=20).

^b^OM: Original items of "FF-MedAd" questionnaire in modified form (e.g., shortened, linguistically corrected; n_total_=10).

^c^OMW: Reinstatement and modification of items from the original version of "FF-MedAd" questionnaire in "FF-MedAd-R" questionnaire (n_total_=6).

^d^OW: Reinstatement and unmodified use of an item of the original "FF-MedAd" questionnaire version in the "FF-MedAd-R" questionnaire to create the new dimension “Drug intake in public” (n_total_=1).

^e^I: Creation of new items (n_total_=43) and dimensions (n_total_=6) in "FF-MedAd-R" questionnaire on the basis of patient interviews [25].

^f^A: Creation of new items on the basis of the authors content-related considerations (n_total_=6).

^g^Final: Remaining items of the "FF-MedAd-R" questionnaire in the “Full path model” after completion of the data analysis (n_total_=37).

^h^Responses varied from 1 (strongly disagree) to 4 (strongly agree).
